# Benefit of a nurse-led telephone-based intervention prior to the first urogynecology outpatient visit: a randomized-controlled trial

**DOI:** 10.1007/s00192-020-04318-0

**Published:** 2020-05-09

**Authors:** Maria Jimènez Torres, Klara Beitl, Julia Hummel Jimènez, Hanna Mayer, Sonja Zehetmayer, Wolfgang Umek, Nikolaus Veit-Rubin

**Affiliations:** 1 Department of Obstetrics and Gynecology, Medical University of Vienna, Waehringer Guertel 18, 1090 Wien, Austria; 2 Department of Nursing Sciences, University of Vienna, Alser Straße 23, 1080 Wien, Austria; 3 Center for Medical Statistics, Informatics, and Intelligent Systems, Medical University of Vienna, Spitalgasse 23, 1090 Wien, Austria; 4 Karl Landsteiner Institute of Special Obstetrics and Gynecology, Silbergasse 18, 1190 Wien, Austria

**Keywords:** Telephone interview, Referral and consultation, Nurse-led interview, Pelvic organ prolapse, Overactive bladder, Pelvic floor disorders

## Abstract

**Introduction and hypothesis:**

Triage has become a valid tool to reduce workload during the first consultation in a specialized clinic. A nurse-led telephone intervention prior to the first urogynecologic visit reduces visit duration and increases patients’ and physicians’ satisfaction.

**Methods:**

All patients scheduled for their very first visit were recruited. They were randomized into an intervention group (prior contact by a specialized urogynecology nurse) and a control group (no contact). The intervention included a questionnaire about history and symptoms. Patients were prompted to complete a bladder diary. Primary outcome was duration of the consultation; secondary outcomes were patients’ and physicians’ satisfaction with the intervention.

**Results:**

Fifty-five patients were allocated to the intervention group and 53 to the control group with no difference regarding age, BMI, parity, menopausal status and primary diagnosis. Mean duration of the telephone call was 10.8 min (SD 4.4). The consultation was significantly shorter in the intervention group than in the control group (mean difference: 4 min and 8 s, *p* = 0.017). In the intervention group, 79% of the patients found the consultation quality “excellent,” 86% would return, and 77% would recommend our clinic to a relative or friend compared with 68%, 67% and 66%, respectively, in the control group. Physicians were “very satisfied” or “satisfied” with the patient preparation.

**Conclusions:**

A nurse-led intervention reduces the duration of the first uroynecologic consultation and is associated with high patient and physician satisfaction. Further research should evaluate whether it also decreases the number of follow-up visits and further referrals.

**Electronic supplementary material:**

The online version of this article (10.1007/s00192-020-04318-0) contains supplementary material, which is available to authorized users.

## Introduction

Triage and patient-related information preparation have become valid tools in times of overcrowded clinics and resource constraints [1]. Numerous triage tools have been validated worldwide, such as the Australasian Triage Scale (ATS) the Manchester Triage System [MTS], the Canadian Triage and Acuity Scale (CTAS), the Emergency Severity Index (ESI) or the Swiss Triage Scale (STS) [2–6]. These scales represent ready-to-use charts during a clinical setting, and some can be implemented over the telephone. Such telephone triage in particular enables timely delivery of efficient and safe high-quality care, permits patient selection according to the complexity of their condition and can be conducted by nurses [7–9]. Previous studies and reviews have demonstrated that a telephone preparation led by nurses was associated with a redistribution of primary care workload for patients requesting same-day consultations and a reduction in the number of out-of-hours visits by general practitioners [10, 11]. The analysis of telephone triage systems in the UK has demonstrated that nurse-led triage is associated with a reduction in general practitioner contact time but with an overall increase in clinician contact time [9]. For the urogynecologic consultation, a thorough history taking is a crucial but time-consuming task. It includes specific questions about symptoms and signs, specifically related to the pelvic floor, the urinary tract and the bowel function and therefore requires special knowledge by the health care provider. Oliver and colleagues have evaluated the efficacy of a model of healthcare delivery including a chart-based triage of urogynecologic patients by a specially trained nurse prior to the first contact with a physician [12]. In this study 59% of patients did not even require a medical consultation because they had minor conditions. It was also shown that the introduction of a nurse-led triage reduced the number of outpatient visits and the time spent in the clinic. This study aimed to evaluate whether a reduction of the very first visit duration by a previously held specialized nurse-led telephone interview can be confirmed in a different population and whether it improves patient’s and physician’s satisfaction.

## Materials and methods

A randomized controlled trial was conducted with patients randomly allocated to either an intervention group or a control group. The intervention consisted of a telephone interview prior to the scheduled visit, conducted by one specialized urogynecology nurse. A maximum of three attempts to contact patients was undertaken. Once the connection was established, the nurse introduced herself by full name and affiliation and asked for permission to perform the interview. If the time was not suitable, an alternative date and time were agreed on. The interview was semi-structured and included questions about current complaints and the medical history (see Appendix A). As part of the telephone interview, the patient was informed about the expected sequence of diagnostic procedures of the planned visit to the urogynecologic outpatient clinic. The patient was asked to complete and bring with her a bladder diary if applicable and all previous medical reports and relevant radiologic studies.

All patients who were scheduled for their first visit to the urogynecologic outpatient clinic or their first visit after 5 years were recruited. Based on the electronic scheduling list, patients were randomized into the intervention group or the control group, using a randomizing software (RandomAssign©, Michael Hummel Vienna). We excluded patients who were scheduled for a follow-up appointment, who had been seen within the last 5 years and who had not provided their telephone number. For all recruited patients, age, parity, vaginal parity, body mass index (BMI), menopausal status and main diagnosis obtained at the end of the visit were recorded. For statistical purposes, patients were summarized into two diagnostic groups: group 1 with pelvic organ prolapse (POP) and other pelvic floor disorders and group 2 with urinary incontinence.

This study was conducted at a large academic tertiary referral center in Austria. Here, patients present on appointment at the registration desk. After registration, they take a seat in the waiting area. In the waiting area, patients do not have contact with either a nurse or physician. Nurse and physician work together in one single examination room and call in one patient after the other. In clinical routine, the nurse regularly provides a briefing for the physician—based on the available information—before the patient is called from the waiting area into the examination room. This was done in the same way in our study, but for the intervention group, the nurse provided more information, based on the previous telephone-based interview.

Physicians were not blinded to the patients’ allocation to either the intervention or control group. Patients in the control group were not contacted, and the physician did not receive more information as part of the briefing by a nurse. The primary outcome variable was the duration of the clinical visit in minutes, measured from the time when the patient entered the examination room to the time when she left. Secondary outcome variables were patients’ satisfaction with the clinic visit and physicians’ satisfaction with the efficiency of the telephone preparation. Patients satisfaction was measured using the ZUF-8 questionnaire, validated in German. The ZUF-8 is derived from its English original, the psychometrically validated Client Satisfaction Questionnaire CSQ-8[13]. It consists of eight items, each measured on a four-point Likert scale, with a total possible score range from 8 (worst agreement) to 32 (maximum agreement); see Appendix B [14, 15]. Physicians’ satisfaction was evaluated using standardized questions including a question about the estimated time economy of the visit (see Appendix C). For the purpose of the study, the diagnosis was recorded at the end of the clinic visit as stated by the physician in the patient’s chart. The study was approved by the ethics committee of the Medical University of Vienna (no. 1646/2017).

A power calculation yielded a sample size of 49 in each group with at least 80% power to detect a mean difference of 5 min between the intervention and control group. We based the power calculation on a mean visit duration of 30 min, using a two group t-test with a 5% two-sided significance level and a standard deviation of a maximum of 8.7 in each group (nQuery 8, Power and Sample Size Calculation).

To examine differences between groups, descriptive statistics and corresponding explorative tests were generated. For the variables age, body mass index (BMI), consultation time and ZUF-8 total score, mean and standard deviation were calculated, and t-tests for group comparison were performed. For the variables parity and vaginal parity, median, interquartile range (IQR) and a Mann-Whitney test were calculated. For the variables ZUF-8 question scores, main diagnosis and diagnostic groups, counts and percentages are given and chi-square tests or Fisher exact tests were calculated. To examine the consultation time, two multiple linear regression analyses were calculated: In the first regression, the influence of group (intervention or control), parity, age and diagnostic group was analyzed; in the second regression, the variable group was not considered. Both regression analyses were repeated for the target variable mean ZUF-8 score. R (version 3.6.) was used for statistical analysis.

## Results

**Fig. 1:**
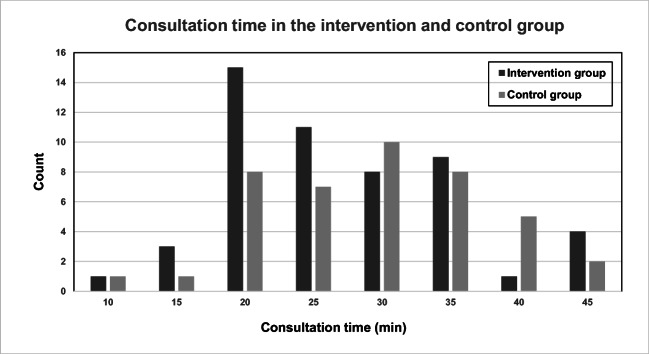
Bar chart showing the distribution of consultation times in the intervention group compared with the control group

Between April and November 2017, we recruited a total of 108 patients scheduled for their very first urogynecologic visit out of a total 190 patients scheduled for an outpatient clinic visit. Fifty-five were allocated to the intervention group and 53 to the control group. Two patients in the intervention group could not be reached by phone, and five patients in the control group did not attend. We therefore analyzed 53 patients in the intervention group and 48 in the control group. Fifity-three telephone calls were successfully performed with a mean duration of 10.8 min (SD 4.4). The two groups did not differ statistically with regard to age, BMI, parity, vaginal parity, menopausal status and main diagnosis (Table 1). Mean duration of the visit in the intervention group was 26 min (SD 8.9) compared with 30.8 min (SD 11.4) in the control group, with a statistically significant difference of 4 min and 50 s (*p* = 0.02) (Fig. 1). For the ZUF-8 satisfaction questionnaire, patients in the intervention group scored a mean of 30.1 (SD 2.7) out of 32 compared with a mean of 29.4 (SD 3.1) out of 32 in the control group (Table 2). Seventy-eight percent of the patients in the intervention group found the quality of the consultation “excellent” (question 1 of the ZUF-8) compared with 71.1% of patients in the control group (Table 2). The distribution of scores per group for all ZUF-8 questions is summarized in Table 2. When asking the physicians about their satisfaction with the patient preparation in the intervention group, 82.69% responded that they were very satisfied. Nine percent of physicians did not believe in a time-saving effect of the intervention, whereas 29% estimated a time-saving effect of 5 min, 36% estimated a time-saving effect of 10 min, and 20% estimated a time-saving effect of > 10 min. In 94% of the situations, the briefing was qualified as helpful.

**Table 1:** Demographics and diagnoses

	Intervention *n* = 53	Controls *n* = 48	*p* value
Age (mean) (SD) (years)	58.58 (12.72)	57.71 (14.50)	0.747*
Body mass index (mean)(SD)(kg/m²)	28.82 (6.77)	26.53	0.127*
Parity (median) (interquartile range)	2 (1–3)	2 (1–3)	0.933**
Vaginal parity (median) (interquartile range)	2 (1–3)	2 (1–3)	0.896**
Hormonal status			0.105***
Premenopause (%) (*n*)	26.4% [14]	41.7% (20)	
Postmenopause (%) (*n*)	73.6% (39)	58.3% (328)	
Main diagnosis			0.503***
Stress urinary incontinence (%) (*n*)	26.4% [14]	14.9% [7]	
Overactive bladder (OAB) (%) (*n*)	22.6% [12]	23.4% [11]	
Mixed urinary incontinence (%) (*n*)	20.8% [11]	27.7% [13])	
Pelvic organ prolapse (POP) (%) (*n*)	24.5% [13]	31.9% [15]	
Other (%) (*n*)	5.7% [3]	2.1% [1]	
Diagnostic groups			0.680***
OAB and urinary incontinence	69.8% (37)	66.0% (31)	
POP and other	30.2% [16]	34.00% [16]	

*Calculated with t-test

**Calculated with Mann-Whitney test

***Calculated with chi-square test

For “parity” and “vaginal parity”: 1 missing value in the intervention group/1 missing value in the control group

For “hormonal status”: 2 missing values in the control group

For “main Diagnosis” and “diagnosis groups”: 1 missing value in the control group

**Table 2:** Scores for the ZUF-8 Questionnaire

		Intervention *n* = 53	Controls *n* = 48	*p* value
ZUF-8 Questions (n) (%)	Score			
Q1: How would you evaluate the quality of the treatment?	1	0 (0)	0 (0)	0.379*
	2	0 (0)	0 (0)	
	3	11 (21.6)	13 (28.9)	
	4	40 (78.4)	32 (71.1)	
Q2: Did you receive the treatment you wanted?				0.497
	1	2 (4.2)	0 (0.0)	
	2	1 (2.1)	0 (0.0)	
	3	12 (25.0)	14 (32.6)	
	4	33 (68.8)	29 (67.4)	
Q3: To what extent did our clinic satisfy your needs?				0.089
	1	0 (0.0)	0 (0.0)	
	2	1 (2.0)	2 (4.3)	
	3	7 (14.3)	14 (30.4)	
	4	41 (83.7)	30 (65.2)	
Q4: Would you recommend our clinic to a friend if he/she needed similar help?				0.394
	1	2 (3.9)	1 (2.2)	
	2	1 (2.0)	0 (0.0)	
	3	8 (15.7)	13 (28.9)	
	4	40 (78.4)	31 (68.9)	
Q5: How satisfied were you with the extent of help you have received?				0.759
	1	1 (1.9)	1 (2.2)	
	2	0 (0.0)	1 (2.2)	
	3	9 (17.3)	10 (21.7)	
	4	42 (80.8)	34 (73.9)	
Q6: Did the treatment you have received help you to cope better with your problem?				1.000
	1	0 (0.0)	0 (0.0)	
	2	2 (4.0)	1 (2.3)	
	3	9 (18.0)	8 (18.6)	
	4	39 (78.0)	34 (79.1)	
Q7: How satisfied were you with the treatment overall?				0.538
	1	0 (0.0)	1 (2.2)	
	2	0 (0.0)	0 (0.0)	
	3	6 (12.0)	13 (28.9)	
	4	44 (88.0)	31 (68.9)	
Q8: Would you visit our clinic again if you needed help?				0.030
	1	0 (0.0)	1 (2.2)	
	2	0 (0.0)	0 (0.0)	
	3	6 (12.0)	13 (28.9)	
	4	44 (88.0)	31 (68.9)	
Total ZUF-8 score (mean) (SD)		30.07 (2.72)	29.41 (3.05)	0.258**

All *p* values were calculated with Fisher’s exact test, except those marked * and **

*Calculated with Pearson chi-square

**Calculated with t-test

The results of the linear regressions are presented in Table 3. Controls have a significantly longer consultation time than patients from the intervention group independently from age, diagnostic group and parity (*p* = 0.028); 6.5% of the variation of consultation time can be explained by the given model (R^2^ = 0.065). POP and other as a diagnostic group is the only independent factor associated with consultation time (*p* = 0.049); 2.5% of the variation of consultation time can be explained by the given model (R^2^ = 0.025). Both the intervention and control groups do not differ for mean ZUF-8 score (*p* = 0.270) independently from age, diagnostic group and parity (*p* = 0.270); 6.6% of the variation of consultation time can be explained by the given model (R^2^ = 0.066). POP and other as a diagnostic group is independently associated with mean ZUF-8 score (p = 0.049); 6.4% of the variation of consultation time can be explained by the given model (R^2^ = 0.064).

**Table 3:** Linear regression models for outcome parameters

Coefficients	Estimate	*p* value
Consultation time		
Group	4.552	*0.028*
Parity	0.563	*0.470*
Age	0.108	*0.164*
Diagnostic group (reference POP)	-4.758	0.034 *
*Multiple R-squared: 0.104, adjusted R-squared: 0.065*		
Mean ZUF-8 score		
Group	−0.641	*0.270*
Parity	−0.293	*0.192*
Age	0.033	*0.139*
Diagnostic group (reference POP)	1.257	*0.047*
*Multiple R-squared: 0.106, adjusted R-squared: 0.066*		

*POP = pelvic organ prolapse*

## Discussion

This study assessed the effectiveness of a nurse-led telephone interview prior to a first consultation in terms of time saving as well as patients’ and physicians’ satisfaction with the intervention. We found a time-saving effect of almost 5 min per patient, which in our setting permits us to see one additional patient per half day in clinic. Our findings indicate that the majority of patients seeking medical help for urogynecologic conditions are satisfied with the provided care when they are prepared by a continence nurse specialist without the need for primary medical contact. The differences were not dependent on demographic factors but varied depending on the diagnosis. Urinary incontinence is indeed a more challenging pathology than pelvic organ prolapse, which may explain the longer consultation times for this particular condition. The return was excellent with only two patients who could not be reached after three attempts at different dates and all reachable patients agreeing to participate. We considered this an indicator for a demand in our patient population for such a pre-consultation preparation.

There was a gap of approximately 5 min between the time gain during medical consultation and the mean duration of the telephone interview in the intervention group. It is important to consider the personnel costs per time unit, which would certainly be higher for medical staff. A telephone interview can be conducted by a single person, whereas, in this setting, an outpatient consultation requires the presence of at least one physician and one nurse. From a patient’s perspective the reduced consultation time will also result in the benefit of an overall shorter length of stay at the clinic. A nurse-led telephone interview appears to accumulate direct and indirect benefits; the time gain for the medical consultation may outweigh the longer duration of the patient’s contact with the institution. However, this hypothesis needs to be tested in a cost-efficiency analysis. Holt and colleagues did not find an association between telephone triage and a reduction in overall clinician contact time in their subgroup analysis of the randomized controlled ESTEEM trial. Although nurse-led triage is associated with a reduction in contact time with the general practitioner, it was associated with an overall increase in clinician contact time [9].

Most patients in the intervention group were positively surprised by the telephone call and appreciated that some fear may have been reduced and that they were informed about and prepared for the details of the upcoming consultation. For several patients, an accompanying translator for the first clinic visit was organized at the time of the telephone interview. The absence of an accompanying translator for the first clinic visit could have necessitated another appointment or at least a prolonged duration of the clinic visit [16]. This is in accordance with a previous study that has shown that patients with limited language proficiency were more likely to receive recommendations for higher acuity care when confronted with a telephone preparation [17].

Physicians who were briefed before the consultation were highly satisfied. Some specific benefits in a urogynecology setting were raised during the study, such as the availability of a bladder diary, which in our practice represents a prerequisite for the prescription of anticholinergic medication. If a bladder diary is not available at the first clinic visit, patients need to be scheduled for another appointment to initiate the therapy.

The strengths of our study are its prospective design and the randomization process. Furthermore, we performed a patient satisfaction assessment using a validated questionnaire, which is commonly used in German-speaking health care institutions. There are several ways to assess patient satisfaction, and such a process represents a significant challenge. Neugebauer and colleagues have highlighted that there is currently no universally recognized definition of the term “satisfaction with health care” [18]. An effective questionnaire-based assessment relies on various factors such as the patient’s first impression of the questionnaire, esthetic aspects, its usability and a limited number of questions. The ZUF-8 fulfills these requirements.

Our study has some limitations. The study question did not permit blinding either patients or physicians to whether a telephone-based interview was performed prior to the visit or not. This creates the possibility for a bias regarding the secondary outcome parameter “patient satisfaction” and “physician satisfaction” and might explain the difference between the intervention and the control group. However, our primary outcome parameter was the objectifiable duration of the clinical visit in minutes. We did not assess the long-term impact of the intervention regarding the number of follow-up visits, proportion of surgical vs. conservative treatment or overall costs per patients. Our findings did not allow conclusions about whether or not shortened consultations would impact the visit’s medical quality.

We did not record the time of the pre-consultation briefing or consider the length of the telephone interview in an overall time-benefit assessment. The intervention under investigation assessed whether valuable physician consultation time in the examination room may be partly substituted by nurse consultation time and in this way increase time efficiency in clinic while at the same time leading to greater satisfaction. The intervention may not decrease overall contact time of the patient with the institution.

In summary, a nurse-led telephone interview prior to a patient’s first urogynecologic outpatient visit is a suitable tool to save time in clinic and improve patients’ and physicians’ satisfaction.

Based on our findings, we hypothesize that training nurses for a telephone interview could decrease the number of follow-up visits and referrals. Also, future studies could address cost efficiency and long-term measurable efficiency of such an intervention.

## Electronic supplementary material

ESM 1 (DOCX 16 kb)

ESM 2 (DOCX 17 kb)

ESM 3 (DOCX 18 kb)
