# Supplementary material for: Benefit of a nurse-led telephone-based intervention prior to the first urogynecology outpatient visit: a randomized-controlled trial
Source: Int Urogynecol J. 2020 May 9;32(6):1489–95. doi: 10.1007/s00192-020-04318-0 (PMC8203547; doi:10.1007/s00192-020-04318-0)
Supplement: Supplementary file 1 — (DOCX 16 kb) [file 192_2020_4318_MOESM1_ESM.docx]

**Appendix A**

**Semi-structured telephone interview* (translation of the German original)**

- Do You have a referral letter from your gynecologist? What does it say?
- What kind of medical problem do You have?
- Do You experience involuntary loss of urine? If YES, do You experience it when coughing, sneezing, laughing? How often? Since when?
- When You experience an urge to empty Your bladder, can You reach the toilet in time?
- If You experience both, loss of urine when coughing, sneezing, laughing and combined with urgency, what bothers You more?
- How many times do You empty Your bladder ?
  during daytime__ after going to bed__
- Do You feel a bulge in Your vagina?

Since when__ How big is it approximately? __cm

- Have You had urinary tract infections? How often per year?
- Do You currently feel a burning or itching during micturition?
- Have You done any therapy for Your problem so far?

If YES, which form of therapy

- Have You undergone surgery in Your life?
- Do You suffer from any medical condition?
- Do You take any medication? If YES, please bring a list of all medications
- Please bring all Your medical reports
- Please bring all Your radiological reports
- Explain bladder diary and send it optionally (mail, fax, e-mail)
- Patient´s social status

Do You live alone? Do You have professional help? From the family?

If necessary, please bring an interpreter !

* filled in by the nurse
