# Supplementary material for: Benefit of a nurse-led telephone-based intervention prior to the first urogynecology outpatient visit: a randomized-controlled trial
Source: Int Urogynecol J. 2020 May 9;32(6):1489–95. doi: 10.1007/s00192-020-04318-0 (PMC8203547; doi:10.1007/s00192-020-04318-0)
Supplement: Supplementary file 2 — (DOCX 17 kb) [file 192_2020_4318_MOESM2_ESM.docx]

**Appendix B**

**ZUF-8 patient satisfaction questionnaire* (English translation of the German original)**

Score 1 2 3 4

1. How would you evaluate the quality of the treatment

excellent___ good___ not so good___ not good___

1. Did you receive the treatment you wanted?

clearly not___ not really___ overall yes___ clearly yes___

1. To what extent did our clinic satisfy your needs?

almost all___ most of them___ only few of them ___ none of them___

1. Would you recommend our clinic to a friend if he/she would need similar help?

clearly no___ I don’t think so___ I think yes___ clearly yes___

1. How satisfied were you with the extent of help you have received?

very unsatisfied___ quite unsatisfied___ mostly satisfied___ very satisfied___

1. Did the treatment help you to cope better with your problem?

it helped a lot___ it helped a little___ it did not help much___ it made it worse___

1. How satisfied were you with the treatment overall?

very satisfied___ mostly satisfied___ quite unsatisfied___ very unsatisfied

1. Would you visit our clinic again if you would need help?

clearly not___ I do not think so___ I think yes___ Clearly yes___

- filled in by the patient
