# Supplementary material for: Benefit of a nurse-led telephone-based intervention prior to the first urogynecology outpatient visit: a randomized-controlled trial
Source: Int Urogynecol J. 2020 May 9;32(6):1489–95. doi: 10.1007/s00192-020-04318-0 (PMC8203547; doi:10.1007/s00192-020-04318-0)
Supplement: Supplementary file 3 — (DOCX 18 kb) [file 192_2020_4318_MOESM3_ESM.docx]

**Appendix C**

**Questionnaire about physician´s satisfaction* (English translation of the German original)**

Pat code ____

- Was the briefing helpful?

Yes__ No__ not applicable (control group)__

- Was the patient correctly scheduled? Yes__ No__
- Did the patient bring the list of medication and was it complete?

Yes__ No__ not applicable__

- Did the patient bring a bladder diary?

Yes__ No__ not applicable__

- Did the patient bring all the relevant medical reports?

Yes__ No__ not applicable__

- Could you make a diagnosis? (no further consultation necessary)

Yes__ No__

- Do you think that the intervention helped to save time?

Yes__ No__ not applicable (control group)__

- If YES, how many minutes would you estimate it saved?

5 minutes__ 10 min__ more than 10 min__

- Did the intervention help for the choice of investigation and exam?

Yes__ No__

- How satisfied were you with the preparation?

very satisfied__ satisfied__ not satisfied__

* filled in by the physician
